# Genetic Mechanisms for Hybrid Breeding in Vegetable Crops

**DOI:** 10.3390/plants12122294

**Published:** 2023-06-12

**Authors:** Hira Singh, Bhallan Singh Sekhon, Pradeep Kumar, Rajinder Kumar Dhall, Ruma Devi, Tarsem Singh Dhillon, Suman Sharma, Anil Khar, Ramesh Kumar Yadav, Bhoopal Singh Tomar, Theodora Ntanasi, Leo Sabatino, Georgia Ntatsi

**Affiliations:** 1 Department of Vegetable Science, Punjab Agriculture University, Ludhiana 141004, India; hira@pau.edu (H.S.); bhallansekhon3249@gmail.com (B.S.S.); rajinderkumar@pau.edu (R.K.D.); rumadevi@pau.edu (R.D.); tarsemdhillon@pau.edu (T.S.D.); 2 ICAR—Central Arid Zone Research Institute, Jodhpur 342003, India; pradeep.kumar4@icar.gov.in; 3 Department of Plant and Environmental Sciences, New Mexico State University, Las Cruces, NM 88003, USA; suman30@nmsu.edu; 4 ICAR—Indian Agricultural Research Institute, New Delhi 110012, India; anil.khar@gmail.com (A.K.); rkyadavneh@gmail.com (R.K.Y.); bst_spu_iari@rediffmail.com (B.S.T.); 5 Laboratory of Vegetable Production, Department of Crop Science, Agricultural University of Athens, IeraOdos 75, 11855 Athens, Greece; ntanasi@aua.gr; 6 Department of Agricultural, Food and Forest Sciences, University of Palermo, 90128 Palermo, Italy; leo.sabatino@unipa.it

**Keywords:** hybrids, self-incompatibility, gynoecism, male sterility, pollen biology, biofortification, vegetables

## Abstract

To address the complex challenges faced by our planet such as rapidly changing climate patterns, food and nutritional insecurities, and the escalating world population, the development of hybrid vegetable crops is imperative. Vegetable hybrids could effectively mitigate the above-mentioned fundamental challenges in numerous countries. Utilizing genetic mechanisms to create hybrids not only reduces costs but also holds significant practical implications, particularly in streamlining hybrid seed production. These mechanisms encompass self-incompatibility (SI), male sterility, and gynoecism. The present comprehensive review is primarily focused on the elucidation of fundamental processes associated with floral characteristics, the genetic regulation of floral traits, pollen biology, and development. Specific attention is given to the mechanisms for masculinizing and feminizing cucurbits to facilitate hybrid seed production as well as the hybridization approaches used in the biofortification of vegetable crops. Furthermore, this review provides valuable insights into recent biotechnological advancements and their future utilization for developing the genetic systems of major vegetable crops.

## 1. Introduction

Hybrids offer significant advantages for enhancing the economic and technological aspects of vegetable cultivation, primarily due to the phenomenon of heterosis, which confers superiority over diverse parent varieties. Hybrids’ exploitation is of the utmost importance in addressing the emerging challenges induced by climate change, including the mitigation of food and nutritional insecurities. Moreover, hybrids provide a powerful tool for breeders to maximize the yield potential of vegetable crops. Heterosis exerts a profound impact on both productivity and quality across a range of vegetable crops, enabling improvements in livelihoods by enhancing productivity and delivering high-quality products and nutritionally superior food options. Leveraging genetic mechanisms to exploit heterosis facilitates enhancing productivity, improving quality (depending on the objective), and reducing seed production costs. The growing interest in heterosis exploitation and the utilization of genetic mechanisms is evident in the intensified investigation of self-incompatibility (SI), male sterility, and gynoecism and their applications in hybrid seed production for several vegetable crops. However, it is important to note that these mechanisms are limited to specific classes of vegetable crops.

Heterosis (also known as hybrid vigor) plays a pivotal role in vegetable breeding, leading to significant improvements in yield, quality, and earliness, which are considered the desirable outcomes when genetically distant parents are used to produce hybrid off-springs in vegetable crops. However, the selection of suitable parents is crucial for achieving these results [1]. Genetic diversity among parents enables the development of inbred lines that exhibit both good general and specific combining abilities, which are then utilized to produce promising hybrids. The genetic system of the parents determines their combining ability and helps to predict selection efficiency. Combining ability analysis helps in understanding gene action and assists in selecting appropriate breeding procedures for quantitative trait improvement. The availability of a larger number of seeds per pollination/cross and the presence of genetic mechanisms such as male sterility (tomato, chili, capsicum, onion, and cole crops), gynoecism (cucurbits, particularly cucumber and bitter gourd), and self-incompatibility (cole crops) offer ample opportunities not only to exploit heterosis but also to drastically minimize the cost of hybrid seed production. Advancements in techniques such as transgenics, gene editing, QTL mapping, genomics, and genome-wide association studies (GWASs) are being employed to predict heterosis in different vegetable crops [2,3,4,5,6] and tobacco (Zejun). However, the existing published research on the genetic mechanisms and the role of genes in defining important plant characteristics is limited. Therefore, studying plants at the gene level and subsequently applying suitable breeding methods is necessary to fully exploit heterosis.

## 2. Heterosis in Vegetable Crops

The development of a commercially viable system for producing hybrid seeds has had a profound impact on the modern scientific understanding of crops and the agricultural industry.In 1914, George Harrison Shull, an American botanist and geneticist, while working with corn at Cold Harbor, New York, coined the term “Heterosis” [7,8], which means the superiority in the performance of F_1_ hybrids over two mated inbreds. During the second decade of the twentieth century, Hayes and Jones [9] first suggested the exploitation of hybrid vigor in cucumber (*Cucumis sativus*). Notably, the development of the commercial eggplant F_1_ hybrid performed in Japan in 1925 marked a significant milestone for hybridization attempts [10,11]. Four classes of vegetables are reported as per the use of heterosis [12]. Initially, the commercial adoption of hybrids was limited due to high production costs. However, the growing availability of published data on the significant heterosis observed in different vegetable crops has motivated breeders to not only develop new hybrids but also explore genetic mechanisms to facilitate more efficient and economically viable hybrid seed production.

## 3. Floral Characteristics of Different Vegetable Crops

To establish a successful hybrid breeding program, comprehensive knowledge of floral characteristics, reproductive features, the time of anthesis, and pollination is required. It is crucial for vegetable growers to possess in-depth knowledge of the different flower types (male, female, hermaphrodite, monoecious, dioecious, and so on), the time of anthesis, dehiscence timing, and the reproductive attributes of vegetable crops [10,13,14] in order to successfully implement the genetic mechanisms involved in hybrid seed production. In most vegetable crops, floral opening predominantly occurs during the morning hours, although exceptions occur among certain cucurbit species. Dehiscence varies from longitudinal to transverse, which, in fact, plays an important role in determining the mode of reproduction. Consequently, a thorough understanding of pollen fertility is essential for achieving a proper seed set. Moreover, when unsynchronized flowering occurs, knowledge of pollen storage techniques and their application in pollination can be valuable, greatly enhancing the efficiency and overall quality of seed production. 

## 4. Genes Controlling Floral Traits in Vegetable Crops

A specific class or a combination of classes of genes determines organ identities. Their role in floral development can be explained using the ABCDE model [15,16], which is applicable to both monocotyledonous and dicotyledonous species. This model demonstrates that five classes of homeotic genes, namely, A, B, C, D, and E, govern floral organ identities [17,18]. As per the floral quartet model [19], the development from the first to the fourth level is outlined earlier by Murai [18]. The key roles of different genes in the development of different floral organs have been elucidated by different authors in their respective studies on several vegetables. In angiosperms, as per the ABC model of flower development, petals and stamens are reported to be under the control of B-function genes. Based on this model, Ning et al. [20] isolated PAP3 (a B-class gene) in pepper, which encodes 226 amino acids with high similarity to the MADS-box protein family, with a conservative MADS domain and semi-conservative K domain. Moreover, shriveling of pollen grains was observed after the knockdown of the PAP3 gene through virus-induced gene silencing (VIGS). This eventually led to male sterility, but no effect on petal development was observed. In line with the ABCDE model, Chuan et al. [21] isolated six genes (BraAP2 (an A-class gene), BraAP3 and BraPI (B-class genes), BraAG (a C-class gene), BraSHP (a D-class gene), and BraSEP (an E-class gene) in Chinese cabbage using PCR amplification. Significant decreases in the expressions of A-, B-, C-, D-, and E-class genes during the first to fourth stages, the first to fifth stages, the first to third stages, and all six developmental stages of floral bud development, respectively, in petal-loss plants (the A-16 and A-17 lines) compared with normal plants (A-8) were reported. These results could be exploited by vegetable breeders as a theoretical basis for the future exploration of the underlying molecular mechanisms. The RsMADS gene in radishes and its associated potential functions with a discussion at the molecular level, particularly regarding the mechanisms underlying flowering and floral organogenesis, has been documented by Li et al. [22]. Furthermore, investigations on the evolutionary relationships and expression profiles along with dominant pathways in relation to flowering genes have been performed [23,24], which may help to improve bolting and flowering in *Raphanus sativus* and other Brassicaceae crops. Using the BLAST technique, 142 potential bolting- and flowering-related genes were identified in various flowering pathways, and, furthermore, out of these 142 genes, the isolation and profiling of 7 critical genes was also reported using TA cloning and RT-qPCR analysis. SQUAMOSA, GLOBOSA, DEFICIENS, AGAMOUS, and SEPALLATA1 were the previously defined groups in carrot in relation to MADS-box genes, and five genes, namely, DcMADS1, DcMADS2, DcMADS3, DcMADS4, and DcMADS5, were assigned to them [25,26]. The development of anthers and pollen was attributed to DcMADS3 and DcMADS5, which belong to the B-class and E-class MADS-box genes, identifying the identity of stamens. Stamens were reported to be completely replaced with carpels when the DcMADS3 gene was down-regulated in the homeotic flowers of carpeloid cytoplasmic male sterility (CMS)-type carrot [25]. Furthermore, the SEP1 gene arbitrates the behaviors of the B- and C-organ identity genes [27,28,29]. The genes controlling flowering and reproduction were assigned to six linkage groups on nine carrot chromosomes as Vrn1 (early flowering habit), cola-locus (male and female organ differentiation defects), DcMADS3, DcMADS5, and Rf1 (restoring petaloid CMS) and were mapped onto LG2, LG4, LG5, LG7, and LG9, respectively. The mapping of the Vrn1 and Rf1 genes was reported by Alessandro et al. [30], while the rest of the genes were assigned to different linkage groups by Budahn et al. [26]. Moreover, a well-saturated map for carrot, incorporating the findings from the aforementioned studies, was developed by the latter. These studies could help breeders to develop male sterile lines in carrot, with the ultimate goal of utilizing the same system for efficient hybrid seed production. 

Similarly, in the case of cucurbitaceous vegetables, particularly in the case of melon and cucumber, male and female flower production is reported to be under the control of various genes. ACS11, the limiting enzyme of ethylene biosynthesis, which is reported to control female flower development, is encoded by the androecy gene. Male and female plants are under the control of ACS11 expression. ACS11 expression leads to female plants while its loss-of-function mutants produce male plants. CmACS11 and CmW1P1 (the male-promoting gene/inhibitor of carpel development) play major roles, and their combination leads to artificial dioecy, while on an individual level, the former represses the expression of the latter to control the development and coexistence of male and female flowers in monoecious species [31]. The mutation of CmWIP1 leads to a gynoecious phenotype in melon [32]. Conversely, in the case of cucumber, CRISPR/Cas9 can be utilized to exploit genetic mechanisms due to its low transformation efficiency [33]. In line with this, an improved transformation protocol for cucumber has been developed, and the generation of a gynoecious cucumber line using the CRISPR/Cas9-mediated mutagenesis of CsWIP1 has been well documented [34]. Upon modification from CmWIP1 to CsWIP1, a rapid increase in the development of gynoecious inbred lines was observed in cucumber, and this was utilized for hybrid seed production. A huge difference in the number of male flowers was observed with the CsWiP1 mutant when compared with the wild type. The new knowledge of genes governing floral characteristics has allowed vegetable breeders to develop new, more stable, and climate-resilient male sterile and gynoecious lines to facilitate hybrid seed production. In the case of highly self-pollinated crops such peas as peas, wherein the keel acts as a restriction against crossing work, the down-regulation of genes governing keel formation may open new opportunities to develop hybrids more efficiently and at a lower cost. Even in the case of cross-pollinated crops in which it is difficult to emasculate the flowers, converting them to petaloid forms could help to facilitate hybrid seed production. In cucurbits, developing gynoecious lines using CRISPR/Cas9 opens new ventures.

## 5. Pollen Biology and Development

Information regarding pollen behavior has gained popularity among breeders as it assists their breeding plans accordingly. Complicated flower biology, sex forms such as dioecy, unsynchronized male and female flowering, and poor and irregular flower production altogether limit wide hybridization. Therefore, the improvement of vegetables through conventional breeding approaches is constrained especially in the case of yam, etc. [35,36]. Pollen storage is considered an effective and versatile approach to overcome these limitations. Pollen behavior can be modified when subjected to different temperatures. There are also reports of enhanced pollen viability under varied storage conditions. The stored pollen, with enhanced viability, can be utilized to facilitate pollination processes, particularly in cases of unsynchronized flowering. Reproduction in angiosperms was reported to be highly selective [37], with female tissues identifying pollen from identical species and rejecting pollen from different species. These processes enable breeders to utilize self-incompatible (SI) systems for hybrid seed production, particularly in the case of Brassicas. The role of high temperatures in breaking these SI systems has been well documented. The effects of temperature on pollen development and SI systems in different vegetable crops have been thoroughly studied and compiled. In the case of tomato, several reports on pollen storage and pollen viability are available. The highest efficiency for fruit set, fruit weight, and the number of seeds per fruit in the case of stored pollen was observed in one-day-stored pollen [38], two-days-stored pollen [39], and nine-days-stored pollen [40]. Similarly, pollen viability was enhanced with three to four days [41], ten days [42,43], and five days of storage at room temperature and seven days in refrigerated storage [40]. Furthermore, the effect of high temperature in relation to pollen viability has also been extensively investigated. High temperatures, i.e., at a day temperature of 32 °C and a night temperature of 26 °C, reduce pollen viability and the number of pollen grains per flower in tomato [44]. This has been associated with alterations in carbohydrate metabolism during anther development. Decreased starch and sugar concentrations in mature pollen have been identified as possible causes for the reduction in pollen viability [44]. Reduced fruit set, fruit weight, and seed number per fruit at high temperatures have been well documented [45,46,47]. In the case of hot pepper, pollen could be stored for up to 47 days when cryopreservation was applied [48], which is a method that has been considered relevant by several researchers [49,50]. Alexander et al. [51] also claimed that Capsicum pollen could retain its viability and fertility for more than 42 months under cryopreservation. Pollen samples of yam retained their germination capacity even after 2 years when subjected to “wet-cold” conditions at −80 °C [52]. In cucurbits, pollen tube growth in watermelon was observed to be highest at 35 °C and lowest at 15 °C [53]. Increasing the temperature from 10 to 32°C resulted in an elevation in the pollen tube growth rate [54]. Sugiyama et al. [55] studied the storage of seedless watermelon pollen and its effect on several characteristics. Storing pollen beyond 1 month in dehydrated ethyl acetate (as an organic solvent) with the temperature maintained at −20 °C has been suggested. Silica gel stored in a sealed container at 5 °C has been found to retain pollen germination on an artificial medium for up to 20 days. In the case of melon, maximum pollen germination and pollen tube growth were observed at 30 °C and 35 °C [56]. Eggplant pollen storage at low temperatures (with the maximum and minimum at −30 °C and −20 °C, respectively) resulted in higher germination percentages [57]. Different chemicals have been explored for eggplant pollen storage, with benzene being more reliable than acetone and chloroform. Freeze-dried pollen (at −60 °C) exhibited the best germination [57]. The trinucleate type of pollen in most Brassicas creates difficulties in handling, storage, and in vitro conditions [58]. Particularly in the case of CMS, poor hybrid seed production is often ascribed to inadequate pollination [59,60,61]. Various pollen viability tests and strategies to overcome inadequate pollination in carrot, cauliflower, and onion have been well documented by Brown [62]. Maintaining the availability of pollen possessing desirable traits from pollen banks represents an efficient and reliable method to improve hybrid seed production.

## 6. Genetic Mechanisms

Various strategies have been proposed for hybrid seed production based on emasculation and pollination procedures. One of the most promising strategies for producing cost-effective seeds with minimal impact on seed purity is the utilization of genetic mechanisms. These genetic mechanisms, namely, self-incompatibility (SI), gynoecism, and male sterility, have proved to be reliable and economically viable options and, therefore, have gained high popularity among breeders. By leveraging these genetic mechanisms, improved yields and desirable traits can be achieved, seed purity can be concomitantly insured, and the need for labor-intensive emasculation and manual pollination can be reduced.

### 6.1. Self-Incompatibility (SI)

Self-incompatibility (SI) refers to a genetically controlled mechanism of certain plant species that prevents self-pollination [63]. It involves the rejection of pollen from the same plant by the pistil, promoting out-breeding and maintaining genetic diversity. This phenomenon was first described by Koelreuter in 1764 [64] and is reported to occur in a wide range of vegetable crops such as cabbage, cauliflower, tomato, etc. Darwin [65] discussed SI for the first time, while significant information on genes and gene products regarding SI trait expression was made available by Dodds et al. [66]. This mechanism implies the inability of viable and functional pollen grains to fertilize ovules carrying similar S-alleles and the inhibition of self- and sib fertilization in a few plant species. This mechanism is particularly evident in cole crops such as *Brassica oleracea* [67], which exhibit a highly self-incompatible system. Extensive reviews and many research reports have explored SI’s fundamental and genetic aspects in both flowering plants and vegetable crops [68,69,70,71,72,73,74,75,76,77,78,79,80,81]. Herein, attempts are made to cover the history of SI, its genetics, the factors involved in SI breakdown, the methods for detecting SI, and its commercial exploitation.

#### 6.1.1. History of Sporophytic Self-Incompatibility (SSI)

Earlier studies have investigated the SI mechanism in cabbage [11,82] and its association with the gametophytic system. Consequently, it was assumed that incompatibility mechanisms or systems that are controlled by several alleles belong to the gametophytic (GSI) and sporophytic (SSI) types of self-incompatibility and are restricted to heterostylic species. Firstly, the SSI system was reported by Hughes and Babcock (1950) in *Crepis foetida*, while Gerstel [83] documented it in *Parthenium argentatum*. Bateman [69,84,85] further elucidated the sporophytic nature of SI in all members of the Brassicaceae family, a finding that has since been corroborated by various researcher groups [86,87,88] studying *Brassica oleracea*.

#### 6.1.2. Genetics of SSI

The discovery of SSI resulted in the production of a huge number of S-locus alleles. In particular, 30 S-locus alleles in *Brassica rapa* [89] and more than 50 in *Brassica oleracea*, 30 to 40 S-alleles in Brassicaceae [90], and more than 100 S-haplotypes in *Brassica oleracea* and *Brassica rapa* have been documented by different authors. Classical genetic analysis categorized the *Brassica* S-alleles into 2 groups based on their phenotypic effect, i.e., with high allele activity placed high on the dominance scale (0 to 10 pollen tubes develop per self-fertilized stigma) and low allele activity considered to be recessive (10 to 30 pollen tubes develop per self-fertilized stigma). Furthermore, genetic analysis revealed that the S-locus cysteine-rich (SCR) protein and S-locus receptor kinase (SRK) were responsible for SI reactions in Brassicas [91,92,93]. The pollen coat protein S-cystine-rich/‘S’ Protein 11 and the stigma-specific S-receptor kinase (SRK) encoded by two tightly linked polymorphic ‘S’ genes have been reported to regulate SI [94]. Other mechanisms governing SI include the requirement for ARC1, U-box proteins in the *Brassica* pistil [95], the involvement of two separate determinate genes [81], and intriguing molecular lock and key mechanisms [96]. The studies on the regulation systems of SI provide valuable insights for breeders and could pave the way for new research avenues at the molecular level. 

Gene action studies related to the SSI system have proved helpful to breeders in many ways. Dominance, reverse dominance, co-dominance, and competitive interactions were among the main gene actions reported by different authors in their respective studies. Thompson [97] suggested that dominance and an independent relationship were possible in a self-incompatible plant with heterozygous S-alleles (SaSb) with sporophytic control in the pollen and stigmas. In this respect, all four types (viz., type I, II, III, and IV) were reported to be present in marrow-stem kale [98] and at extremes in Brassicas [99]. Furthermore, intermediate variations were reported to cause the complete weakening of both S-alleles [99]. These allelic relationships between S-alleles, dominance in the pollen, and independent action in the stigmas were observed to be the most common gene actions, as reported by Tatebe [100] in radish, Adamson [88] in cabbage, and Richards and Thurling [86] in *Brassica campestris*. However, the co-dominance of alleles (varying in number) in both the stigmas and pollen was observed by Sampson [101] in Broccoli, Sampson [102] in radish, Haruta [103] in Chinese cabbage and turnip, Hoser-Krauze [104] in cauliflower, and Negi [105] in cabbage. Moreover, competitive interaction was observed by Thompson and Taylor [106] in kale, Lawson and Williams [107] in *B. oleracea*, and Hadj-Arab et al. [108] in cauliflower. Haruta [103] observed the dominance of the same allele in both the pollen and stigmas, while Litzow and Ascher [109] discussed the case of reverse dominance in *Brassica* spp. Sampson [110] summarized co-dominant, dominant, and incompletely dominant relationships with reference to SI in broccoli, while reports of dominant and co-dominant relationships in Brassicaceae were documented by de Nettancourt [111]. Kakizaki et al. [112] reported the linearly dominant relationship of pollen in *Brassica campestris*. In relation to S-haplotypes, the S-locus revealed a dichotomy in sequence, and all class-I haplotypes were reported to show dominance over all class-II haplotypes in the determination of pollen specificity [113], while Hatakeyama et al. [114] observed that the relationship between two S-haplotypes may be co-dominant/ dominant or recessive in the determination of the phenotypes of the stigmas and pollen in an S-heterozygote. Shiba et al. [115] working with *Brassica rapa* and *Brassica oleracea* demonstrated that dominant/recessive relationships are regulated at the mRNA level. Understanding the gene action related to SI is highly valuable, as S-alleles with higher dominance are likely to exhibit minimum selfs and sibs in hybrid seeds. Such knowledge can greatly assist breeders in identifying and isolating S-allele homozygotes/heterozygotes.

#### 6.1.3. Breakdown of SI

Various genetic and environmental factors can contribute to the production of self-seeds in an SI population, which is a phenomenon known as pseudo-compatibility or breakdown of incompatibility. Genetic processes involved in this phenomenon include the presence of weakly active S-alleles in the style [84], competitive interaction between S-alleles [107,116], and the predominance of recessive alleles [107,117,118]. Additionally, this phenomenon may also be attributed to the genetic consequences of inbreeding shifts [119]. Other factors, such as the temperature [120,121], humidity [71,121], flowering stage [122,123], and age of the flower [107], have been documented to affect the level of SI in Brassica species. Thermally aided pollination, steel-brush pollination, and electric-aided pollination have been suggested by Roggen and Van Dijk [124], Roggen and Van Dijk [125], and Roggen et al. [126] as methods to break SI, respectively. In conclusion, SI decreases with increasing temperature and humidity, and compatibility tends to increase toward the end of the flowering season. These environmental and climatic factors might interact to induce a multidimensional situation in field conditions. Therefore, proper care needs to be taken when designing breeding programs using SI germplasm.

#### 6.1.4. Detection and Maintenance of SI

Various methods for the detection of SI have been proposed by different researchers. These include conventional breeding approaches [87,98,99,127], pollen grain staining and stigma darkening [84,85,98], the seed setting/fertility index [70,128], marker genes [70], serological methods [74], fluorescence microscopy [129,130], molecular approaches [131,132,133], and polyacrylamide-gel isoelectric focusing. Test crosses, diallel crosses, and reciprocal crosses are the major approaches that have been suggested in relation to conventional breeding programs. Among other approaches, the seed setting or fertility index method has been widely employed. However, fluorescence microscopy has gained popularity among breeders in the 21st century. With the advancements in molecular markers, various molecular approaches such as PCR-RFLP, PCR-CAPS, RAPD, BSA, and SCAR markers have been utilized for the characterization of different S-alleles. RFLP aids in the identification and isolation of S-alleles in homozygous lines, while CAPS is deployed to characterize different alleles at the SI locus. BSA and RAPD have proven useful for identifying markers linked to the SI gene in self-incompatible and self-compatible near-isogenic lines. SCAR markers have shown potential for improving SI lines and accelerating marker-assisted selection processes in SI hybrid breeding programs.

#### 6.1.5. Commercial Exploitation of SI

Pearson [134] first proposed the use of SI for hybrid seed production in *Brassica*. Subsequent studies on SI feasibility in various crops of the *Brassica* group, such as cabbage [82,88], broccoli [135], and kale [136], were conducted. Further research evaluated SI lines and hybrids in cabbage [137], cole crops [138], Brussels sprout [122], and cauliflower [104,139,140,141,142,143,144,145,146]. In the case of *Brassica*, single, double, three-way, and triple crosses were preferred for hybrid seed production. Double, three-way, and triple crosses proved to be more cost-effective than a single-cross hybrid seed production method. However, intensive preparatory breeding work to isolate, maintain, and increase the SI lines as well as test their combining ability was required. Moreover, the lack of uniformity in hybrids developed with SI is also a major problem. To overcome these problems, strategies such as the use of isogenic lines as parents [82,147] for economical and uniform hybrid production and approaches involving high temperatures, double pollination, end-of-season pollination, the use of 3–5% carbon dioxide (CO_2_) gas [148,149], and the use of 3% sodium chloride (NaCl) spray have been proposed by various researcher groups [150,151] to prevent labor-intensive and time-consuming bud pollination and to overcome inbreeding problems. Asexual or vegetative propagation, specifically meristem culture under in vitro conditions, is another method to resolve these problems. To counter sibling problems, the use of parental lines with synchronized flowering, similar morphologies, and pollination via stored pollen has been suggested. The careful selection of inbreds with high levels of SI and minimal morphological differences is of significant importance. Type-IV S-allelic interaction (co-dominance between the pollen and stigmas) is essential for three- and four-way crosses because a single cross is reciprocally incompatible with both of its parents (a homozygous inbred), and, in consequence, any intended selfs in the single cross would not diminish the genetic purity of the three- and four-way crosses [99]. Moreover, the role of honeybees as pollinators in SI cannot be overlooked [152] as their preferences for particular inbreds may lead to the ultimate hybrids. Though many problems with SI systems have been encountered, solutions have also been proposed, and both private-sector companies, as well as public institutions, continuously exploiting this system worldwide (Table 1). This extensive literature review could help young scientists who are engaged in or are planning to work with SI systems.

### 6.2. Gynoecism

In vegetable crops, SI and male sterility systems are mainly confined to the Brassicaceae and Solanaceae families. There are reports of such systems in other families, particularly Cucurbitaceae. The Cucurbitaceae family has been endowed with highly variable sex types and is one of the plant families characterized by predominantly unisexual flower production. Through evolutionary processes, the sex forms in cucurbits emerged as monoecious, gynoecious, andromonoecious, androgynoecious, and gynomonoecious types [41,155,156]. Among these forms, the gynoecious flowering habit has been exploited for the hybrid breeding of cucurbits, particularly in cucumber and bitter gourd (Table 2). Therein, labor operations such as the pinching of staminate flowers and pollination were reported to be economically feasible [157,158].

#### 6.2.1. Genetics

Well-documented studies have been conducted on the inheritance of gynoecism (femaleness) in cucumber [41,80,159]. The interaction between the mutated gene (recessive for gynoecious expression) and the dominant intensifier for the female sex gene (In-F) was reported to govern the expression of pistillate or femaleness [159]. Similarly, in melon, the interaction between two genes, *a* and *g*, governs stable gynoecism [156]. Furthermore, gynoecism is reported to be under monogenic dominant control [160] in cucumber, while a single recessive gene (*gy-1*) controls gynoecism in bitter gourd [155,161,162]. On the contrary, partial dominant control in the case of bitter gourd has been observed by Iwamoto and Ishida [163]. Attempts were also made to study gene action in bitter gourd gynoecious hybrids through generation mean analysis [162] and duplicate epistasis. Transgressive segregants were found in advanced generations for most traits. Furthermore, significant additive and non-additive gene effects were observed for traits related to earliness, suggesting the utilization of reciprocal recurrent selection (RRS) or bi-parental mating (BPM) to enhance these traits. Non-additive gene action was found to be significant for fruit length, the number of fruits per plant, fruit weight, and yield, indicating that heterosis breeding is an ideal option for achieving higher gains in these traits.

#### 6.2.2. Biotechnological Advances

Closely linked SSR markers to the gynoecious (F) locus, namely, SSR13251 and UW020605 at 1.0 and 4.5 cM, respectively, were identified in cucumber by Jat et al. [164]. A total of 17 markers were placed on chromosome 6 along with the F locus covering a total distance of 100.4 cM and were used for genotyping and linkage map analysis. Single dominant gene control was reported specifically in the gynoecious genotypes GPC-1 and PPC-2. Furthermore, the use of specific markers and dominant gene control in marker-assisted backcross breeding for transferring gynoecious character into horticulturally desired cultivars was recommended. Similarly, an inter simple sequence repeat (ISSR) marker linked to gynoecism in bitter gourd was identified. DBGy-201 was used as a gynoecious line, and, subsequently, 24 plants were screened using 200 RAPD and 28 ISSR markers. The primer (AC)8T amplified a 1000 bp fragment specific to gynoecious plants. This marker enables the identification of gynoecious plants in just 35–40 days after sowing, offering a cost-effective approach. Collaborative efforts in the exploration of gynoecism in cucurbit breeding programs should be encouraged, leading to more efficient and effective strategies.

#### 6.2.3. Commercial Exploitation

The induction of male flowers in cucumber gynoecious lines for commercial-scale hybrid breeding with the application of growth regulators has been reported by Robinson [165]. Before this study, Peterson and Anhder [166] proposed the use of silver nitrate as an alternative to gibberellic acid due to its inconsistent effects on male flower induction. The inhibition of ethylene action by silver ions was identified as the main mechanism for the above-mentioned induction [167]. However, due to the phytotoxic effects of silver ions, the use of silver thiosulphate was recommended instead [168]. The use of silver nitrate (AgNO_3_) (50–100 ppm) or silver thiosulfate (25–50 ppm) in gynoecious plants at the 2–3-leaf stage has been suggested to stimulate the production of staminate flowers. Furthermore, the use of 6 mM of silver nitrate has been suggested in bitter gourd to induce hermaphrodite flowers [162]. However, environmental factors significantly influence the performance of gynoecious lines. Optimal temperature conditions above 30 °C and photoperiods of up to 12 h have been proposed as desirable, with the photoperiod having no impact at high temperatures. It should be noted that the expression of the same gene can vary depending on the environmental context, necessitating the careful consideration of gene–environment interactions in gynoecism studies.

### 6.3. Male Sterility

With respect to male sterility, genic male sterility (GMS), cytoplasmic male sterility (CMS), and cytoplasmic genetic male sterility (CGMS) systems have gained popularity among breeders for hybrid seed production (Table 2). GMS has been exploited in a restricted number of crops such as tomato, chili, etc., while CMS systems play a key role in the Brassicaceae family, particularly in cabbage and cauliflower. CGMS systems are employed in hot pepper, onion, and carrot. TGMS (temperature-sensitive genetic male sterility) and PGMS (photoperiod-sensitive genetic male sterility) have also been reported in several vegetables but are not extensively exploited. The search for novel male sterility–fertility restoration systems that overcome transgenic issues is also underway. Reviews on the history and genetics of male sterility are already available [169,170,171,172,173,174,175,176,177,178,179,180,181,182,183]. Furthermore, research reports discussing both the genetic and molecular levels of different kinds of male sterilities (GMS, CMS, and CGMS) are available for tomato [184,185,186], brinjal [187,188], hot pepper and sweet pepper [189,190,191,192,193,194,195,196,197,198], okra [199], muskmelon [200,201,202], watermelon [203,204], radish [193,205,206,207,208,209,210,211,212,213,214,215,216,217,218], onion [183,219,220,221,222,223,224,225,226,227,228], garlic [229], carrot [230,231], cucumber [232], bean [233,234,235], broccoli [236], cauliflower [171,237,238,239], Brussels sprout [147], sugar beet [240], chives [241], and cabbage [242,243]. The non-availability of efficient systems for the identification of male sterile plants in a genic male sterile system limits its utilization. Morphological markers such as the potato leaf marker, green stem and anthocyanin-less stem in tomato, anther color (purple or yellow) and shriveled anther size in hot pepper, glabrous seedling in muskmelon, glossy foliage in Brussels sprout, and purple stem pigmentation in cabbage have been reported to aid in hybrid seed production.

**Table 2:** Crops and genotypes with respect to self-incompatibility, male sterility, and gynoecism in vegetable crops.

Crop	Hybrid/Line	System	Remark	Reference
Tomato	‘ms33 IPA’ as female parent	GMS	Genic male sterile line with exerted stigma saved 54.4% of time.	[244]
Hot pepper	CH-27	GMS	Multiple-disease-resistant hybrid obtained 55% extra yield compared withCH-1.	[245]
	CH-3	GMS	Out-yielded recommended varieties by 80–100%; multiple-disease-resistant.	[246]
	CH-1	GMS	Out-yielded recommended varieties by 80–100%; multiple-disease-resistant.	[246]
Okra		GMS	First report in the world.	[199]
		GMS	Evaluated hybrids and reported high heterosis for yield/plant.	[247]
	Arka Nikita	GMS	New GMS-based okra F1 hybrid.	[248]
Muskmelon	Punjab Hybrid	GMS	High-yielding and disease-resistant.	[249]
	Punjab Anmol	GMS	High-yielding and disease-resistant.	[200]
Cauliflower	Ogu1A, Ogu2A, and Ogu3A	CMS	Ogura type.	[250]
Kale	MS-91, MS-51, MS-11, and MS-110	CMS	Ogura type.	[177] [251]
Chili	KashiSurkh and Kashi Early from CCA-42-61 and PBC-473, respectively	CGMS	Suitable for green as well as dry fruit.	[252] https://iivr.icar.gov.in/hybrid-kashi-early (accessed on 26 July 2022)
	Arka Meghana, Arka Sweta, and Arka Harita	CGMS	Arka Harita; tolerant to powdery mildew and viruses.	[252]
Onion	Arka Kirthimaan and Arka Lalima	CGMS	IIHR; tolerant to purple blotch, basal rot diseases, and thrips.	[253]
	Hybrid-63 and Hybrid-35	CGMS	IARI, New Delhi.	[177]
	DOGR Hy—7	CMS	ICAR-DOGR, Pune.	[254]
	DOGR Hy—50	CMS	ICAR-DOGR, Pune.	
	DOGR Hy-1, DOGR Hy-2, DOGR Hy-3, DOGR Hy-4, DOGR Hy-5, and DOGR Hy-8	CMS	ICAR-DOGR, Pune.	
Carrot	Pusa Nayanjyothi and Pusa Vasuda	Petaloid CMS	Pusa Nayanjyothi and Pusa Vasuda are the first temperate and tropical CGMS-based hybrids, respectively.	[255]
Cucumber	CGN-19533, CGN-20256, CGN-20515, CGN-20953, CGN-20969, CGN-21585, CGN-22930, Gyne-5, and Pusa Sanyog	Gynoecism	Based on crosses among these lines, researchers suggested to exploit heterosis breeding commercially for developing high-yielding, quality parthenocarpic gynoecious hybrids.	[256]
	GBS-1	Gynoecism	Inbred could be exploited for yield and earliness.	[160]
	PPC2, GPC1	Gynoecism	SSR markers closely linked to the F locus will be useful in marker-assisted backcross breeding for transferring gynoecious trait into horticulturally desirable varieties.	[164]
Bitter gourd	Gy263B	Gynoecism	Gynoecism in Gy263B is under the control of a single, recessive gene.	[155,157]
	DBGy-201	Gynoecism	Used to develop Pusa Hybrid 3 and 5.	[257]
Cauliflower	Pusa Hybrid-2 and Pusa Karthik Sankar	SI	Field-resistant to downy mildew.	[258]

## 7. Biotechnological Advancements

### 7.1. Novel Male Sterility–Fertility Restoration System

Singh et al. [259] developed a novel male sterility and fertility restoration system to facilitate hybrid seed production in crops where seeds are of high economic importance. This system, besides being biologically safe, also enables the production of pure hybrids. Moreover, it could help to overcome the limitations of previously available systems such as Barnase–Barstar systems, chemical methods, and cytoplasmic (CMS) and genic male sterility (GMS) mechanisms. The system involves the functional complementation of a TATA-box mutant TGTA promoter and a TATA-binding protein mutant3 (TBPm3) in an expression-cassette-based system along with modifications for regulatory control. By combining the long hypocotyl in the Far-Red1 fragment (HFR1NT131) with TBPm3 (HFR1NT131-TBPm3), the tapetum-specific constitutive photo-morphogenesis 1 (COP1) is expressed in the male parent, leading to the suppression of BECLIN1 and, concomitantly, to normal tapetal development and fertility restoration. COP1-HFR1 interaction and the COP1-mediated degradation of the TBPm3 pool (HFR1NT131-TBPm3) are the main forces working behind this system. It could be efficiently utilized in hybrid seed production programs for various vegetable crops, as suggested by the authors, and could act as an alternative to existing systems.

### 7.2. Marker-Assisted Selection (MAS) for C-GMS Line Development

Two CMS-specific SCAR markers were developed to distinguish N-cytoplasm from S-cytoplasm and AFLP markers linked to the fertility restorer gene Rf [260]. The CAPS marker (E-AGC/M-GCA122) linked to the *Pr* locus that is related to the partial restoration of fertility in CMS was also reported in chili pepper (*Capsicum annuum* L.) [261]. The SCAR marker, CRF3S1S, was highly efficient (100%) at differentiating restorers from maintainers. The use of CRF3S1S allows the unambiguous detection of restorers in untested ABLs and/or any germplasm, saving both time and resources. 

Globally, S-type cytoplasm in onion is always preferred due to its stability in diverse environments and being genetically governed by a single gene, the *Ms* gene [221]. PCR-based specific markers identifying the cytoplasm have been testified. Regarding *Ms* locus identification, marker-assisted selection made it possible with RFLP [223], CAPS [227], SCAR [262], SNPs [263], and other PCR-based markers [226,264,265,266]. Recently, new primers for the cytoplasm [225,267] and the *Ms* locus [264,265] are being utilized for the identification of the cytoplasm and the *Ms* locus [183,268,269,270]. Although they are reported to be in complete linkage disequilibrium with the *Ms* locus, new PCR markers linked to the *Ms* locus are still being reported. For the identification of the cytoplasm, two PCR markers specific to onion cytoplasm, viz., accD, an indel marker [267], and MKFR designed on a chimeric gene, orf725 [225], were utilized. For *Ms* locus determination, two PCR markers reported for the restorer-of-male-fertility (*Ms*) locus, namely, AcSKP1 [265] and AcPMS1 [264], were employed. Khar et al. [228] concluded that approximately 95% of Indian onion cultivars possessed a homozygous recessive *Ms* locus. Furthermore, they observed that the limitations of PCR-based markers for the identification of the *Ms* locus still exist and need to be addressed by utilizing new PCR markers.

## 8. Biofortification in Vegetables through Hybridization

The fortification of key vitamins, antioxidants, and micronutrients has been highlighted in recent advances in conventional plant breeding. Traditional breeding methods such as variety evaluation in available germplasm, pre-breeding, selection, hybridization, heterosis breeding, mutation breeding, and polyploidy breeding are the most widely used biofortification approaches that offer long-term, cost-effective alternatives to transgenic and agronomic approaches [271]. However, the process of developing nutrient-rich varieties through breeding approaches requires the biochemical categorization of target nutraceuticals at crucial stages [272].

Traditional breeding methods, excluding mutation and polyploidy breeding, require sufficient genetic variation for traits such as carotene and other useful carotenoids, iron, zinc, and other minerals [273]. Limited availability of genetic variation in the gene pool poses challenges in selecting appropriate breeding strategies. Overcoming this limitation involves incorporating genetic material from wild relatives and introducing desirable traits into commercial cultivars such as beans and peas, which exhibit significant variations in Fe and Zn contents (a 6.6-fold difference) [274]. Apparently, tubers generally display reduced genotypic variation [275]. Wild relatives of vegetables that are rich in quality traits and are used for breeding purposes to enhance the nutrient contents of popular varieties are listed in Table 3. These wild relatives serve as donor parents in crossbreeding with recipient lines possessing desirable agronomic features, resulting in hybrid varieties of high nutritional value.

In cases where nutrient-rich donor parents are not cross-compatible with the recipient parents, we can alternatively transfer the genes/QTLs related to the target nutrient into a compatible variety to produce inbreds or pure lines that can be further used for hybridization to develop desirable biofortified hybrids. Information regarding the genes/QTLs for nutritional quality is given in Table 4. For traits that can be phenotypically assessed in an early generation, such as the color-related genes in cauliflower, the transfer of genes can be relatively straightforward. However, in the case of other qualitative traits such as essential minerals, visual inspection is not feasible at an early stage. In such cases, molecular breeding techniques such as marker-assisted selection (MAS) can be used for the successful transfer of the target gene/QTL. This is a method of selecting desirable individuals in a breeding scheme based on DNA molecular marker patterns instead of, or in addition to, their trait values. It is a valuable tool for plant breeders, enabling a more efficient selection of desired crop traits. MAS allows the identification of individuals carrying the trait of interest without relying solely on their phenotype in the early generation [276]. In recent years, efforts have been focused on identifying markers that are strongly associated with genes/QTLs for nutritional quality, which are similar to the markers linked to the gene responsible for orange color in cauliflower.

The International Food Policy Research Institute, in collaboration with the International Center for Tropical Agriculture and the CGIAR, has started the Harvest Plus program to breed biofortified staple food crops [299]. The major goal of this program is to boost the availability of vitamin A and micronutrients, including iron and zinc, in Asian and African staple food crops such as wheat, rice, maize, cassava, pearl millet, beans, and sweet potato [300]. Its purpose is to develop staple food crops that have higher levels of bioavailable essential minerals and vitamins to improve the micronutrient status of target populations, especially resource-poor individuals in developing nations [301]. The Bio-cassava Plus project was also created to increase the nutritional quality of the cassava crop [302]. There are some commercial hybrids with enhanced nutraceuticals have been developed in different vegetables by different institutions (Table 5).

## 9. Future Prospects

Although significant efforts have been made by researchers and institutions to develop hybrids through the utilization of genetic mechanisms, the real economic potential of these mechanisms remains untapped. Moreover, new and innovative traits should be introgressed into the backgrounds of strong and stable SI, CMS, and CGMS lines that can be further utilized for the development of hybrids in vegetable crops. This becomes particularly important in the face of climate change, wherein the development of hybrids, particularly for off-season production, with better adaptability, a significant number of nutraceuticals, and the ability to impart multiple resistance to biotic and abiotic stresses is of utmost importance. Molecular approaches can play a vital role in strengthening genetic mechanisms. This includes the development of novel fertility–sterility restoration systems and the exploitation of genetic tools to down-regulate specific genes [303] for improving male sterility systems. The genomic mapping of SI alleles can help derive mechanisms to enhance and strengthen SI lines, while CRISPR/Cas9 technology can be employed to develop robust and stable gynoecious lines in cucurbits. Furthermore, the use of RNAi and TILLING approaches is advocated for vegetable improvement [304]. Implementations in which such systems have been exploited include the development of multiple-disease-resistant hybrids by Dhaliwal et al. [245], the evaluation of CMS lines that are rich in antioxidants and flavonoids, the use of CRISPR-Cas9 by Hu et al. [34], and the development of a novel sterility–fertility restoration system by Singh et al. [259].

## 10. Conclusions

Significant progress has been achieved in vegetable hybrid breeding in the last decades through the exploitation of genetic mechanisms such as self-incompatibility and male sterility. Gynoecism has also recently gained popularity, and the application of this system in cucurbitaceous vegetable crops holds great potential. The integration of genomic tools, particularly the molecular markers that are practically feasible for the identification of GMS at the seedling stage, will help to enhance its scope. It will open new avenues to exploit monogenic recessive male sterile lines in several vegetable crops. Marker-assisted breeding, particularly the mapping of nutritional-, abiotic-, and biotic-stress-resistant genes, could be helpful to introgress such genes into newly developed genetic-mechanism-based hybrids.

## Figures and Tables

**Table 1:** Reports of lines with high and stable self-incompatibility (SI).

Crop	Lines	Remarks	Reference
*Brassica oleracea* var. *botrytis* L.	Aghani, Pusi, and Hisar-1	Complete SI	[139]
	74-6C	High and stable SI	[145]
	Early Kunwari (September maturity)	High and stable SI	[145]
*Brassica oleracea* var. *italica*	Palam Samriddhi, Calabrese Sutton, BR-76018, DPGB-5, EC-10356, Broccoli Green Head, BI-80167, BI-80336, and Punjab Broccoli 1	SI	[153]
*Brassica rapa*	Kal-22, Kal-3, and Ch1-504	High level of SI	[154]

**Table 3:** Crop wild relatives with high-quality features useful for breeding.

Crop	Wild Relatives/Landraces/Varieties/Accessions	Nutrient
Tomato	*S. pimpinellifolium* and Caro Red (Rugers×*S. hirsutum*)	Vitamin A
	Caro Rich, F-7045, VRT-35, CGT, and VRT-5	Beta carotene
	High-pigment mutants (*hp*), Crimpson (*og*), Pusa Rohini,	Lycopene
	*S. pennellii* IL6-2, IL7-2, and	Phenolics
	*S. pennellii* IL12-4	Ascorbic acid
	*S. chilense* and atroviolacium (*atv*) from *S. cheesmaniae*	Anthocyanin
Chili	*C. annuum* var. IC: 119262(*CA2*), Bayadaggi (kaddi), and	Ascorbic acid
Paprika	KTPL-19	Capsanthin
Cucumber	*Xishuangbanna* gourd (*C. sativus* var. *Xishuangbananesis*)	Beta carotene
Muskmelon	Honeydew 32 and	Ascorbic acid
	Canary yellow	Flavons (Naringenin chalcone)
Spine gourd	*Momordica dioca*	Protein
	*M. chochinchinenesis*	Lycopene
Bitter gourd	DRAR-1 and DVBTH-5	Beta carotene
	DRAR-1 and DVBTG-5	Ascorbic acid
Sweet potato	Resisto, Zambezi, and Chiwoko	Beta carotene
Cassava	UMUCASS 44, UMUCASS 45, and UMUCASS 46	Vitamin A
Broccoli	*Brassica villosa*	Glucosinolates

**Table 4:** Genes/QTLs for nutritional quality in some vegetables.

Crop	Traits	Gene/QTL	References
Tomato	Vitamin C	*Vtc 9.1* (higher vitamin C)	[277]
	Fruit color/carotenoids	*B* (*Beta*) (yellow fruits)	[278]
		*ogc* (*old gold-crimson*) (higher lycopene content)	
		*Del* (*Delta*) (orange fruits)	[279]
		*r* (*yellow flesh*) (yellow fruits)	[280]
		*t* (*tangerine*) (orange fruits)	[281]
		*hp-2* (*high pigment*) (higher lycopene content)	[282]
		*Dg* (*darkgreen*) (higher lycopene content y-uncolored epidermis)	[283]
		*Apricot* (*at*)	[284]
	Anthocyanins	*Anthocyanin fruit* (*Aft*) (anthocyanin in the skin and outer pericarp)	[285]
			[286]
		*Atroviolacium* (*atv*)	[287]
		*Aubergine* (*Abg*)	[288]
Chili	Fruit color	*Y* (yellow fruit color)	[289]
		*C2* (orange fruit color)	[290]
		*A* (purple fruit color)	[291]
Brinjal	Anthocyanin	*fap10.1*	[292]
Onion	Bulb color	*P* (pink color)	[293]
Cauliflower	Curd color	*β-carotene accumulation*/*Or* gene	[294]
		*Pr* (high anthocyanin content)	[295]
Kale	Leaf color	*BoPr* (purple leaf)	[296]
Carrot	Carotenoids	*PSY*	[297]
Watermelon	Lycopene	*LCYB*	[215]
Broad bean	Tannins	*zt-1* and *zt-2* (reduced tannins)	[298]

**Table 5:** Some nutraceutical-enhanced hybrids of vegetable crops.

Crop	Variety	Description	Chief Nutrient Element	Nutrient Content		Institute Developed
Carrot	Pusa Nayanjyoti	First orange-colored temperate carrot hybrid	β-carotene	1.89 mg/100 g	7.55 mg/100 g	IARI, New Delhi
	Pusa Meghali	Selection from Local red × Nantes Half Long cross	β-carotene	1.89 mg/100 g	11571 IU/100 g	IARI, New Delhi
Sweet potato	Sree Kanaka	Tubers with dark orange flesh color Inter-varietal hybrid	β-carotene	2.0–3.0 mg per 100 g	9–10 mg/100 g FW	CTCRI (2017)
Tomato	Punjab Red Cherry	Following pedigree selection, interspecific hybridization between *Solanum esculentum* and *Solanum pimpinellifolium*	Lycopene	2.57 mg per 100 g of fresh weight	4.9 mg per 100 g of fresh weight	PAU (2015)
Potato	Kufri Neelkanth	Developed through hybridization and selection method	Anthocyanin	Negligible	>100 µg/100 g fresh wt	CPRI, Shimla
Cassava	Sree Visakham	A hybrid between a local cultivar and a Madagascar variety	Carotene	-	466 IU 100 per gm	CTCRI, Thiruvanantpuram

## Data Availability

Not applicable.
